# Type 1 diabetes management and hospitalisation in the over 25’s at an Australian outer urban diabetes clinic

**DOI:** 10.1186/s12902-022-01057-9

**Published:** 2022-05-31

**Authors:** Shivani Patel, Celine Farkash, David Simmons

**Affiliations:** 1 Department of Diabetes & Endocrinology, Campbelltown Hospital, Campbelltown, NSW Australia; 2 Macarthur Clinical School, Western Sydney University, Campbelltown, NSW Australia; 3 School of Medicine, Western Sydney University, Campbelltown, NSW Australia

**Keywords:** Type 1 diabetes mellitus, HbA1c, Management audit, Benchmarking, Diabetes complications, Diabetes in pregnancy

## Abstract

**Aims:**

To describe clinic management and referral pathways among adults with type 1 diabetes (T1D) aged > 25 years attending a public outpatient diabetes service.

**Methods:**

Retrospective cohort study of people with T1D aged > 25 years seen by endocrinologists in one Australian urban public outpatient in 2017. Electronic and paper medical records were reviewed using a dataset adapted from the UK National Institute for Health and Care Excellence 2015 guidelines.

**Results:**

Among the 111 people with T1D (mean age 41 ± 13 years, 55% men, mean body mass index 27.1 ± 5.6 kg/m^2^), mean HbA1c was 8.1 ± 1.9% (66 ± 19 mmol/mol) (lower than the Australian National Diabetes Audit: 8.5%/69 mmol/mol) with 25.5% meeting the guideline target of < 53 mmol/mol (7.0%). Most people had seen a diabetes educator (80.2%) or dietitian (73.0%) and had complication screening. Complication rates were high (nephropathy 20.4%, retinopathy 27.4%, peripheral neuropathy 30.1%, ischaemic heart disease/acute infarction 10.5%). Overall, 27% of referrals occurred following an acute inpatient admission or emergency department presentation and 13% for management of diabetes in pregnancy.

**Conclusions:**

A high proportion of people with T1D accessed public specialist care either during pregnancy or after a largely avoidable acute glycaemia-related hospital presentation. Subsequent care was in line with national specialist standards. This area has a “wait for acute event” rather than “complication prevention” model of care, associated with under-referral to the local multidisciplinary specialist service. Understanding how widespread this model of care is, and ways to reduce its prevalence, are urgently required.

**Supplementary Information:**

The online version contains supplementary material available at 10.1186/s12902-022-01057-9.

## Introduction

Type 1 diabetes (T1D) is a lifelong autoimmune condition which requires long-term specialist team involvement [1]. The condition is associated with a significant increase in morbidity and mortality, with an estimated loss in life expectancy at birth of 12.2 years compared with the general population [2]. Individuals with T1D are admitted to hospital more frequently than those without diabetes including for both diabetes (e.g. diabetic ketoacidosis acidosis (DKA), renal disease) and non-diabetes (e.g. infections) related conditions [3]. This poses a considerable financial burden on the individual and healthcare system.

With good self-management and quality care (including structured diabetes education) much of the acute hospital and emergency department (ED) attendance related to hyper- and hypoglycaemia can be avoided [4, 5]. Long term hyperglycaemia related complications can be prevented by intensive monitoring and control of glycaemia by multidisciplinary teams [3, 4]. The risk of these macrovascular and microvascular complications can be further reduced through blood pressure and lipid management [6–9]. Such treatment standards have been defined by guidelines for the management of T1D [10, 11]. These guidelines emphasise the importance of effective self-management and patient education in achieving treatment targets, as well as regular complication screening as a means of secondary prevention of complications [1].

In Australia, the 2017–18 Australian National Diabetes Audit (ANDA) provides the most recent data on the current quality of care for all adults with T1D in Australia [12, 13]. They reported a national mean glycated haemoglobin (HbA1c) for people > 18 years of 8.5% (69.4 mmol/mol). Several studies of individual Australian young adult/transition clinics have described the challenges of managing hyperglycaemia among young adults aged < 25 years [14, 15]. However, few studies have looked explicitly at those beyond the young adult years in Australia, investigating what happens to such people after the additional issues for many young adults have “settled down” and maturity is well in place, or among those diagnosed after the age of 25 years [16, 17].

The aim of this study was to describe the outpatient management of T1D adults aged > 25 years at a public diabetes service and to assess how this relates to Australian population-based data, national and international guidelines. A prior audit among young adults had shown a high rate of readmissions for DKA [14], and hence we were particularly interested in hospital admission rates to provide an objective measure of the effectiveness of the management programs.

## Methods

This was a retrospective audit of people with T1D aged > 25 years who saw an endocrinologist in the adult multidisciplinary diabetes clinics (also attended by a diabetes educator and diabetes dietitian) at Campbelltown Hospital, New South Wales between 1/1/2017 and 31/12/2017. The age threshold was chosen as those aged up to 25 years are seen within the paediatric (0–18 years) and transition (18–25 years) diabetes services. Individuals were identified through the endocrinologist, educator and dietitian patient records which were kept separately. People with latent autoimmune diabetes in adults (LADA) and type 2 diabetes were excluded. Pregnant women were included in this study to match their inclusion in the national audit. HbA1c, blood pressure and body mass index (BMI) were, however, reported for both the whole study population and with pregnant women excluded as targets vary in pregnancy. Electronic and archived (paper) medical records were reviewed by SP/CF. The clinic data collection tool was based upon the UK National Institute for Health and Care Excellence (NICE) T1D audit tool [11] adapted for local use. The most recent weight, body mass index and blood pressure were recorded. Data relating to Emergency Department (ED) presentations and hospital admissions between 2010 and 2017 were collected from the electronic records. Diabetes related admissions were defined as those for an acute glycaemic event (e.g. DKA), ischaemic heart disease, cerebrovascular disease, peripheral vascular disease, diabetic foot disease, nephropathy, depression and/or infection. Non-diabetes related admissions were defined as admissions unrelated to acute or chronic complications of diabetes. Attendance at the structured education program, Dose Adjustment for Normal Eating (OzDAFNE), was recorded following introduction into the service in 2016. Two courses had been rolled out by the time of data collection. The data were collated into an Excel spreadsheet, and subsequently coded and transferred to IBM SPSS. The proportion of the population seen in the clinic catchment was calculated from the local government agency T1D data in the National Diabetes Services Scheme (NDSS) Diabetes map (https://map.ndss.com.au/#!/: updated January 2019). Comparison was made with guideline recommendations and national [12, 13, 16] population-based reports. International reports were used where national data were lacking [18, 19]. Care standards (e.g. treatment targets and complication screening) were based on the most recent Australian guidelines available at the time of the audit [10]. No lipid targets were specified in the T1D guidelines so type 2 diabetes guidelines were used [20].

Data were analysed using IBM SPSS Statistics Software, Version 25. Frequencies and percentages are shown for categorical variables, with mean ± standard deviation (SD) or median (range) shown for continuous variables. Pearson chi-squared tests and one-way analysis of variance (ANOVA) were performed to compare categorical and continuous characteristics between groups. Statistical significance was defined as p-value < 0.05 and 95% confidence intervals were used.

## Ethics

The project was approved as a Quality Improvement Project by the Campbelltown Hospital Quality and Safety Office, under the ethical framework overseen by the South Western Sydney Local Health District Human Research Ethics Committee (SWSLHD HREC) (CT01_2017, 1/07/2017).

## Results

**Fig. 1:**
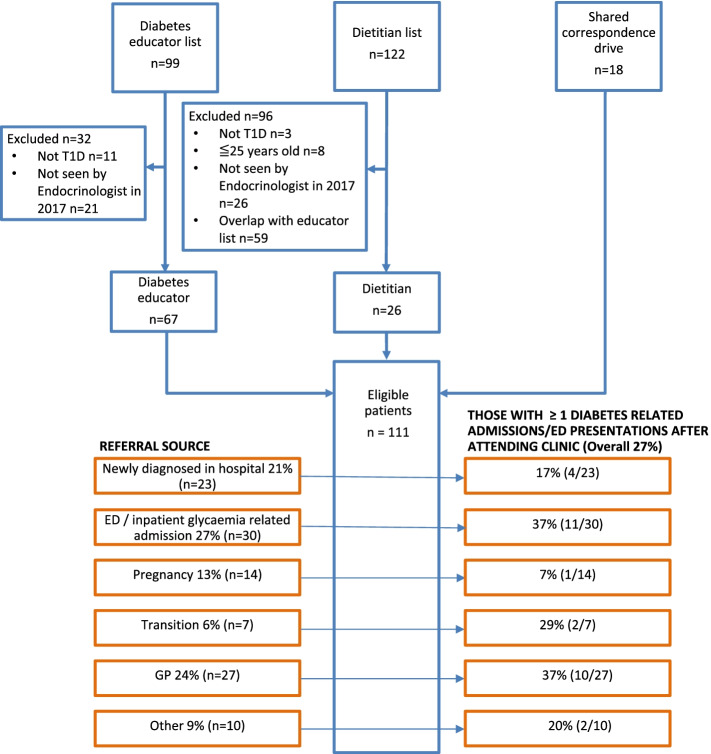
Patient administrative sources, referral source and proportions by referral source with subsequent hospital admission or emergency department presentation. *T1D* type 1 diabetes, *ED* emergency department, *GP* general practitioner

Figure 1 shows the source of the 111 people with T1D who had seen an endocrinologist. Based on the NDSS diabetes map, 137/1330 (10.3%) of the catchment T1D population were seen by the service. Figure 1 also shows that the majority of people were referred to the clinic from the emergency department (27%), after diagnosis in the hospital (21%), once pregnant by the obstetricians (13%) or from the hospital transition clinic (6%). The others were mainly referred by the general practitioner (GP) (24%). Excluding people seen with newly diagnosed T1D in hospital, the majority (63%) of people seen in the clinic had a disease duration greater than 11 years (median). Of these, 30/88 (34%) entered the service following an acute ED presentation or inpatient admission, 14/88 (16%) entered the service once pregnant and 27/88 (31%) were referred by the GP after a mean duration of 22 years of T1D. For the chart review, only one set of paper records was missing. Table 1 shows the characteristics of the cohort. The mean age was 41.4 ± 12.7 years, of whom 55% were men and 77.5% were born in Australia. The majority of people (81.1%) were treated with a basal-bolus insulin regimen and 16.2% used an insulin pump. Overall, 55.5% of people used carbohydrate counting to calculate their bolus insulin dosing. The median diabetes duration was 11 years (range 0.1—54.0). Twenty-five of eighty people (31.2%) with documented smoking status were smokers.

**Table 1:** Participant characteristics

Characteristic	*N* = 111
Age (years)	
Mean ± SD	41.4 ± 12.7
Males	61 (55)
Born in Australia	86 (77.5)
Indigenous	2 (1.8)
Pregnant^i^	14 (12.6)
Duration of diabetes (years)	
Mean ± SD	15.2 ± 13.5
Median (range)	11 (0.1–54.0)
Age first attended clinic (years)	
Mean ± SD	38.5 ± 13.0
Median (range)	37.0 (17–68)
Duration of clinic attendance (years)	
Median (range)	2 (0–10)
Missed 1 or more appointments in last 18 months	48 (43.2)
Smoking status (*n* = 80)	
Non/Ex-smoker	55 (68.8)
Current smoker	25 (31.2)
Mode of insulin treatment	
Basal-bolus	90 (81.1)
Pump	18 (16.2)
Other (BD mixture, basal-only, oral anti-hypoglycaemic agents^ii^)	3 (2.7)
Method of insulin dosing (*n* = 110, 1 not using insulin)	
Carbohydrate counting with variable insulin dosing	61 (55.5)
Fixed Insulin dose	46 (41.8)
Neither (estimating dose)	3 (2.7)

Data reported as N (%) unless otherwise stated. Where information was not available for all people with T1D, numbers in parentheses in the first column indicate the number of people with the data recorded. i. Pregnancy documented at any time in 2017. BD = twice-daily

Table 2 compares the metabolic levels and screening rates achieved with the guideline targets and national cohort data. While the HbA1c of the majority of people was above the national target of < 7%, the mean was below that achieved in ANDA. Almost all people with T1D (94.8%) were self-monitoring blood glucose at least daily, with a quarter testing ≥ 4 times/day as recommended, and a further 46.9% with the precise daily frequency unspecified. Overall, 13.6% of people experienced hypoglycaemia (< 4 mmol/L) on a daily basis. The mean systolic and diastolic blood pressures were within guideline target range, below the national mean, with 49.5% both systolic and diastolic blood pressure within target. The mean total cholesterol (TC) and low-density lipoprotein (LDL) cholesterol were both above target range, with < 20% achieving the lipid targets. Table 3 shows that recording metabolic, renal and foot assessments and attendance with educator or dietitian were generally the same or better than ANDA/UK National Diabetes Audit (UKNDA). However, BMI recording, podiatry and psychologist attendance were less frequent than ANDA. A relatively high proportion had evidence of nephropathy, peripheral neuropathy and macrovascular complications.

**Table 2:** Metabolic standards

Metabolic Outcomes and Control	*N* = 111	^i^ANDA^(12)^	Target^(10, 20)^
Age (years)	41.4 ± 12.7	55.4 ± 17.8	
HbA1c (*n* = 110) %	8.2 ± 1.7	8.5 ± 1.8	< 7.0%
mmol/mol	66 ± 19	69 ± 19	
HbA1c excluding pregnant women^ii^ (*n* = 96) %	8.4 ± 1.7		
mmol/mol	68 ± 19		
HbA1c in pregnant women^ii^ (*n* = 14) %	7.0 ± 0.9		< 6.0–7.0%^iii^
mmol/mol	53 ± 10		
Rate of hypoglycaemia (*n* = 88)			Never
Never – n (%)	18 (20.5)		
≤ 1/week – n (%)	26 (29.5)		
2—6 days per week – n (%)	32 (36.4)		
≥ 1/day – n (%)	12 (13.7)		
Blood Pressure SBP/DBP (mean ± SD, mmHg)			
Whole cohort	122 ± 16 / 72 ± 11	126 ± 17 / 75 ± 10	< 130/80
Excluding pregnant women (*n* = 84)	123 ± 16 / 72 ± 10		
Pregnant women (*n* = 13)	113 ± 15 / 69 ± 12		
SBP < 130 – n (%)	59 (60.8)		
DBP < 80 – n (%)	65 (67.0)		
SBP < 130 and DBP < 80 – n (%)	48 (49.5)		
On antihypertensive therapy – n (%)	25 (22.5)		
BP (on antihypertensive therapy)	132 ± 16 / 72 ± 11	136 ± 19 / 76 ± 11	
BP (not on antihypertensive therapy)	119 ± 15 / 72 ± 10	122 ± 14 / 74 ± 10	
Lipids (mmol/L)			
Total cholesterol (*n* = 94)	4.7 ± 1.1	4.8 ± 1.2	< 4.0
LDL cholesterol (*n* = 83)	2.8 ± 0.9	2.6 ± 1.0	< 2.0
HDL cholesterol (*n* = 81)	1.4 ± 0.4	1.5 ± 0.5	≥ 1.0
Triglycerides (*n* = 90)	1.2 ± 0.9	1.4 ± 1.8	< 2.0
Total cholesterol < 4.0 (*n* = 94) – n (%)	18 (19.1)	22.1%	
LDL cholesterol < 2.0 (*n* = 83) – n (%)	14 (16.9)	24.9%	
HDL cholesterol ≥ 1.0 (*n* = 81) – n (%)	72 (88.9)	91.7%	
Triglycerides < 2.0 (*n* = 90) – n (%)	80 (88.9)	84.2%	
On lipid lowering therapy – n (%)	31 (27.9)	30.0%	
Total Cholesterol (on lipid lowering therapy)	4.5 ± 1.4	4.6 ± 1.4	
Total Cholesterol (not on lipid lowering therapy)			
LDL Cholesterol (on lipid lowering therapy)	4.8 ± 0.9 2.5 ± 1.0	4.9 ± 1.1 2.3 ± 1.0	
LDL Cholesterol (not on lipid lowering therapy)	2.8 ± 0.8	2.8 ± 0.9	
BMI (kg/m^2^) (*n* = 56)	27.1 ± 5.6	26.8 ± 5.8	18.5—24.9
Healthy weight (18.5–24.9) – n (%)	23 (41.1)	44.4%^v^	
Overweight (25–29.9) – n (%)	19 (33.9)	32.1%	
Obese (≥ 30) – n (%)	14 (25.0)	23.5%	
BMI (kg/m^2^) excluding pregnant women (*n* = 47)	27.2 ± 5.3		
Healthy weight (18.5–24.9) – n (%)	19 (40.0)		
Overweight (25–29.9) – n (%)	12 (25.5)		
Obese (≥ 30) – n (%)	16 (34.0)		

Data reported as mean ± SD unless otherwise stated. Where information was not available for all people with T1D, numbers in parentheses in the first column indicate the number of people with the data recorded. i. ANDA includes people aged ≥ 18 years. Whilst ANDA looks at all types of diabetes, all reported ANDA results are specific to people with type 1 diabetes. ii. Pregnancy documented at any time in 2017. iii. Australian guidelines state that for type 1 diabetes in pregnancy, a target HbA1c of < 6.0% is desirable but unless this can be achieved safely, a conservative target of < 7.0% is recommended. Thus, a target of < 6.5% is often used clinically [10]. iv. 1 g proteinuria was calculated as UACR ≥ 70 [21]. v. Combined underweight and healthy weight categories (BMI < 25 kg/m.^2^). vi. No subjects in this cohort were underweight with BMI < 18.5. *ANDA* Australian National Diabetes Audit, *UKNDA* United Kingdom National Diabetes Audit, *HbA1c* glycated haemoglobin, *SBGM* self-blood glucose monitoring, *SBP* systolic blood pressure, *DBP* diastolic blood pressure, *LDL* low-density lipoprotein, *HDL* high-density lipoprotein, *BMI* body mass index

**Table 3:** Processes of care

Glycaemia and Complication Risk Factor Screening	This study	ANDA^(12, 13)^	UKNDA^(18, 19)^	Target^(10, 22)^
Proportion of people with information recorded/assessed in the last 12 months				100%
Frequency of SBGM	51 (45.9)			
HbA1c	110 (99.1)		84.8%	
Rate of hypoglycaemia	88 (79.3)			
Blood pressure	97 (87.4)		90.6%	
Total cholesterol	94 (84.7)		80.2%	
BMI	56 (50.4)		81.7%	
Microvascular Complication Screening				
UACR recorded in last 12 months	91 (82.0)		51.3%	
Duration of diabetes ≥ 2 years (*n* = 93)	81 (87.1)			100%
Retinal screening in last 24 months	94 (84.7)			
Duration of diabetes ≥ 2 years (*n* = 93)	82 (88.2)			100%
Retinal screening in the last 12 months (*n* = 111)	84 (75.7)	74.0%^i^		
Foot Complication Screening				
Feet examined in past 12 months	88 (79.3)		74.1%	100%
Saw a podiatrist in past 12 months (*n* = 66)	18 (27.3)	48.2%		
Macrovascular Complication Screening				
Screening (presence/absence recorded in notes)				
IHD	95 (85.6)			100%
MI	93 (83.8)			100%
CVA	91 (82.0)			100%
Complication Prevalence				
UACR (mg/mmol creatinine) categorisation (*n* = 91)				
Normoalbuminuria (F: < 3.5, M: < 2.5)	68 (74.7)			
Microalbuminuria (F: 3.5–35, M: 2.5–25)	12 (13.2)			
Macroalbuminuria (F: > 35, M: > 25)	11 (12.1)			
Nephropathy reported (*n* = 98)	20 (20.4)			
Retinopathy reported (*n* = 95)	26 (27.4)	23.3%		
Peripheral neuropathy reported (*n* = 93)	28 (30.1)	14.7%		
Macrovascular complications				
IHD (*n* = 95)	10 (10.5)		1.3%	
MI (*n* = 93)	6 (6.4)		0.5%	
CVA (*n* = 91)	3 (3.3)		0.5%	
Education				
Sick day plan discussed	79 (71.2)			100%
Ketone monitoring discussed	82 (73.9)			100%
“5 to drive” discussed (*n* = 107 as 4 people were not driving)	83 (77.6)			100%
Hypoglycaemia management plan discussed	107 (96.4)			100%
Taught how to carbohydrate count	86 (77.5)			
Basal bolus insulin (*n* = 90)	67 (74.4)			100%
Insulin pump (*n* = 18)	18 (100)			
Attended diabetes educator in last 12 months	89 (80.2)	82.5%		100%
Attended dietitian in last 12 months	81 (73.0)	49.2%		100%
Attended OzDAFNE course	8 (7.2)			
Psychological wellbeing & support				
Depression	16 (14.4)	27.7%		
Attended psychologist/psychiatrist in last 12 months	4 (3.6)	19.5%^ii^		

Data reported as N (%). Reported results are specific to people with type 1 diabetes unless otherwise stated. i. Refers to proportion of people with all types of diabetes who attended an optometrist or ophthalmologist. ii. Proportion of people who attended a psychologist in the last 12 months (psychiatrist attendance not reported). *ANDA* Australian National Diabetes Audit, *UKNDA* United Kingdom National Diabetes Audit, *SBGM* self-blood glucose monitoring, *HbA1c* glycated haemoglobin, *BMI* body mass index, *UACR* urine albumin-creatinine ratio, *F* female, *M* male, *IHD* ischaemic heart disease, *MI* myocardial infarction, *CVA* cerebrovascular accident, *OzDAFNE* Australian Dose Adjustment For Normal Eating (structured education course)

**Table 4:** Acute versus non-acute referrals to multi-disciplinary type 1 diabetes service

	Acute referral (ED/inpatient/other hospital) *N* = 30	Non-acute referral (GP/transition/other endocrinologist) *N* = 36	*P*-value
Age at diagnosis (years)	30 ± 11	20 ± 12	**0.002**
Duration of T1D (mean) years	13.7 ± 14.8	21.5 ± 12.0	**0.022**
HbA1c (%)	8.5 ± 2.1	7.8 ± 1.9	0.151
Age	43 ± 11.6	42 ± 14.6	0.613
Complications (any)	10/30 (33%)	16/36 (44%)	0.358

Individuals referred during pregnancy (*n* = 14), unclear referral source (*n* = 8) or new diagnosis (*n* = 23) were excluded from this analysis. ED emergency department, GP general practitioner, T1D type 1 diabetes, HbA1c glycated haemoglobin

Figure 1 also shows the proportion of people who had one or more hospital admissions or ED presentations after entering the service by referral source, with the highest proportions amongst those referred following an acute hospital presentation or those referred by their GP.

When comparing those who were referred to the service acutely (i.e. following an acute inpatient admission, ED presentation or referral from another hospital) to non-acute referrals (i.e. those referred by the GP, transition clinic or other Endocrinologist), non-acute referrals were diagnosed at a younger age and had a longer duration of diabetes than those referred acutely (Table 4.)

## Discussion

We studied 111 T1D adults who attended a multi-disciplinary public outpatient diabetes service in 2017 and found that our study population had a lower mean HbA1c and blood pressure compared to national data with high rates of patient education, educator and dietitian involvement. Despite this high quality of care, the prevalence of diabetes complications was high (31%) and 27% had one or more diabetes related admissions while under the service. Many were originally referred following an inpatient admission or emergency presentation due to either acute or chronic diabetes related issues, rather than from diagnosis. Similarly, those referred non-acutely from an outpatient setting tended to already have had their diabetes for many years.

While there are a range of studies of type 1 diabetes in childhood and the transition period [22–24], few exist focussing on those aged > 25 years i.e. from the end of the transition period. Most studies are national/international [12, 16, 17, 25] losing the granularity shown in our own study. As shown elsewhere, despite a suboptimal proportion of people achieving metabolic targets, most metabolic outcomes in our study compared favourably not only with the national standards shown in Tables 2–3, but also with other national and international data shown in Supplementary Table 1 [12, 16, 17, 25]. Among those not meeting blood pressure or total cholesterol targets, few were recorded as being on antihypertensive or lipid-lowering therapy respectively. Furthermore, of those receiving pharmacological therapies, a minority were meeting the metabolic targets [26, 27]. The prevalence of smoking was elevated at 31% compared to 12.7% in ANDA [12]. While this may be skewed by under-reporting of non-smokers among the 31 individuals without smoking status documented, even if all were non-current smokers, this would still indicate a high proportion of smokers (25/111 ie 23%). An alternative explanation is that the smoking is more associated with reduced self-management adherence [28] and/or more associated with co-morbidities [29], both increasing the chance of hospitalisation/referral.

Attendance rates with dietitians and diabetes educators were high, almost 1.5 times those reported in ANDA [13] and not shown in the other studies. This represents the multidisciplinary team (MDT) model of care used as part of best practice internationally [30, 31]. We anticipate rates of education to increase, as more OzDAFNE sessions are now being offered [32]. There were, however, lower recorded attendance rates to podiatrists (despite high rates of foot disease [33]), and mental health professionals, (despite high documented rates of depression). Moreover, it is likely that these rates of depression are under-reported due to poor record-keeping and compounded by high reported rates of psychological distress within the South West Sydney Local Health District, 2.1% above the state average [34]. These lower rates of access to mental health support are common both in Australia and internationally [35–37] and reflect the frequent absence of e.g. psychologists from the diabetes MDT (including this service).

Rates of retinopathy, peripheral neuropathy and macroalbuminuria were all higher than those reported in ANDA [12], including approximately double the prevalence reported for the latter two. Macrovascular complications were not reported for T1D alone in ANDA; however our study found markedly higher rates (6–12 fold) than the UKNDA [19], despite 30% of UKNDA subjects being under 30 years of age. We postulate that these complication rates are a consequence of delayed referral to specialist care.

### Implications for practice

Multidisciplinary care provided by a public service represents an ideal model of care in T1D, which is accessible to all people regardless of financial status and able to be audited and regulated. This is exemplified by the largely tax funded Swedish healthcare system and UK National Health Service, whereby nearly all people with T1D attend multi-disciplinary specialist clinics through hospitals [38]. In Sweden, upon diagnosis of T1D, individuals are registered in diabetes registries and undergo annual quality registration. Under the NHS, it is possible to undertake national audits (e.g. the national diabetes in pregnancy audit [39] and implement and monitor national type 1 diabetes guidance [11]. In contrast, the current Australian healthcare system delivers fragmented, inconsistent diabetes care through a mixed private, public healthcare system. This is compounded by socioeconomic disparities, (e.g. in this catchment area) with rates of hospitalisation of people from low socioeconomic groups in Australia reported to be 1.6 times higher compared to those from higher socioeconomic groups (303 vs 195 per 100,000) [40].

Achieving metabolic targets nationally are also less likely due to less well-defined targets and treatment indications in Australian T1D treatment guidelines [10], in contrast to American [41] and UK guidelines [10, 42]. Whilst Australia has some care standards in place, they are poorly defined, particularly for T1D. This is in contrast to the UK system, which has nine well-defined annual care processes for T1D management, all of which are regularly audited. Furthermore, Australian guidelines lack specific clinical indications for commencing antihypertensive and lipid-lowering therapy in T1D.

Our interpretation of the metabolic outcomes and complication rates shown here is therefore that the while public hospital care reflects best practice, the local population “model of care” has led to a biased clinic population, often with poorly managed diabetes prior to entry into the clinic. This is also demonstrated in the discrepancy between median duration of diabetes (11 years) and median duration of clinic attendance (2 years). Essentially, the pattern seems to reflect a population-based model of care involving referral (or hospitalisation) after complications have developed, rather than a focus on preventative management. One alternative explanation for the concentration of those with complications in the clinic are that they are those attend the private sector until diabetes and/or non-diabetes related co-morbidities lead them to choose to consolidate care at the one site. However, a high proportion of attenders entered acutely and not by referral (i.e. by asking their GP for a referral). Alternatively, attenders may be those unable (e.g. through cost or health literacy issues) or unwilling to self-manage. Factors which may contribute to this pattern include a higher proportion from low socioeconomic groups and lower health literacy in the catchment. However, self-management and metabolic metric were as good, if not better than those in e.g. ANDA. Barriers to care have been minimised through e.g. reduced clinic waiting time for newly diagnosed patients, as referrals are triaged by an endocrinologist. Logistically, access to clinic is convenient with close proximity to public transport (train and bus stations) and readily available patient parking. Such factors are likely to be cost-saving for local patients particularly those with multiple co-morbidities/complications as care is delivered free of charge under Medicare, with ease of access to multiple other specialties and services from one location.

### Strengths / Weaknesses

This is the first Australian study of its kind to show the current referral pathways into a multi-disciplinary public hospital diabetes clinic. Consistent practice standards and patient demographics of this clinic were strengthened by a breadth of data from multiple endocrinologists practicing in this service, thus not solely evaluating one physician’s quality of care.

This single-centre study was limited by a small study population, at a single site and represented a specific sample of people with T1D in an outer urban centre, hence may not be generalisable to the wider Australian outpatient diabetes clinic population. In addition, this study only analysed admissions to one hospital, hence is likely to underestimate admissions particularly in people with care and admissions elsewhere. Further multi-centre studies are needed to better understand the trends/disparities across various Australian outpatient diabetes clinics. As data were collected retrospectively, the medical records were variable with frequent missing data, only those seen by an endocrinologist in 2017 were included and causative analysis could not be performed. Medical records were fragmented and inconsistent. A more structured approach to medical records including use of diabetes registers, may help standardise and improve care and auditing processes. Since conducting this audit, this service has transitioned to electronic medical records and integrated the use of a secure, Australian web-based database (Biogrid Diabetes Database) designed by Endocrinologists to improve data collection and thus facilitate the ability to perform regular clinical care audits.

Thirteen percent of people were referred to the diabetes clinic by Obstetric colleagues. This could bias some of the metabolic outcomes favorably due to stricter targets in pregnancy. To account for this, HbA1c and blood pressure were also analysed with pregnant women excluded and the means remained lower than the national means [12]. These women are generally referred for glycaemic control during pregnancy, raising concerns in relation to whether these women received adequate pre-pregnancy counselling and glycaemic control [43, 44]. Pre-pregnancy planning clinics have since been introduced in this hospital.

## Conclusion

In conclusion, quality of care in this clinic was in line, if not better, than national standards, with high rates of multidisciplinary team attendance and complication screening. This complex cohort illustrates a suboptimal population-based model of care as people with T1D are referred following a potentially preventable glycaemia related hospital presentation, and/or with longstanding diabetes duration. This is likely to reflect under-referral and thus, high rates of complications prior to enrolment in the local specialist service. Further work is needed to identify how widespread this “model of care” is under the Australian and other health systems, the reasons for under-management of T1D outside the public hospital specialist service and how to reduce hospital admissions and long-term complications. 

## Supplementary Information

**Additional file 1:** **Supplementary Table 1.  **Proportion of people with T1D achieving metabolic targets in selected published audits

## Data Availability

The data used in this study are not publicly available due to the sensitive nature of hospital patient data. For further details relating to the data, please contact the corresponding author.

## Abbreviations

T1D: Type 1 diabetes
DKA: Diabetic ketoacidosis
ANDA: Australian National Diabetes Audit
LADA: Latent autoimmune diabetes in adults
BMI: Body mass index
NICE: National Institute for Health and Care Excellence
ED: Emergency Department
OzDAFNE: Australian Dose Adjustment for Normal Eating
NDSS: National Diabetes Services Scheme
SD: Standard deviation
ANOVA: Analysis of variance
GP: General practitioner
UKNDA: United Kingdom National Diabetes Audit
MDT: Multidisciplinary team
