# Supplementary material for: Type 1 diabetes management and hospitalisation in the over 25’s at an Australian outer urban diabetes clinic
Source: BMC Endocr Disord. 2022 May 31;22:143. doi: 10.1186/s12902-022-01057-9 (PMC9158186; doi:10.1186/s12902-022-01057-9)
Supplement: Supplementary file 1 — Additional file 1: Supplementary Table 1. Proportion of people with T1D achieving metabolic targets in selected published audits [file 12902_2022_1057_MOESM1_ESM.docx]

*Supplementary Table 1: Proportion of people with T1D achieving metabolic targets in selected published audits*

| Study and Year | Population | Setting | HbA1c Target  (%) | % Achieving | BP Target  (mmHg) | % Achieving | LDL Target  (mmol/L) | % Achieving | TC Target  (mmol/L) | % Achieving |
| --- | --- | --- | --- | --- | --- | --- | --- | --- | --- | --- |
| Current Study (2017) | T1DM (n=111)  Age: >25y (mean 41.4y) | Sydney-based outpatient diabetes service | <7 | 25.5% | <130/80 | 49.5% | <2.0 | 16.9% | <4.0 | 19.1% |
| ANDA (12) (2017) | T1DM and T2DM  Age: ≥18y (mean 55.4y) | Diabetes centres in Australia | ≤7 | - | ≤130/80 | 50.1% (T1DM and T2DM) | <2.0 | 24.9%  (T1DM) | <4.0 | 22.1%  (T1DM) |
| Single centre audit in Australia (16) (2003) | T1DM (n=96) and T2DM (n=509)  Age: ≥18y (mean 44.4y) | Sydney-based outpatient diabetes service | <7 | 13%  (T1DM) | ≤130/80 | 29% (treated), 60% (untreated)  (T1DM) | <2.6 | 60% (treated), 36% (untreated)  (T1DM) | <5.5 | 63% (treated), 83% (untreated)  (T1DM) |
| UKNDA (18)  (2017-18) | T1DM and T2DM  Age: all^i^ | Diabetes centres in England and Wales | <7.5 | 29.9%  (T1DM) | <140/80 | 74.8%  (T1DM) | - | - | <5.0 | 70.3%  (T1DM) |
| US T1D Exchange (24)  (2016-18) | T1DM  Age: 1-93y^ii^ | US-based endocrine centres | <7 | 21% | - | - | - | - | - | - |
| International T1D registry audit (17)  2010-2013 | T1DM  Age: all^iii^ | International:  19 national, regional population- based or clinic-based registers | <7.5 | <15 years: 15.7-46.4%  15-24 years: 8.9-49.5%  ≥ 25 years: 20.5-53.6% | - | - | - | - | - | - |

i. The UKNA collected data for patients of all ages. However, whilst proportion achieving HbA1c target was reported for all ages, the proportion achieving blood pressure target was only reported for those ≥12 years. ii. The US T1D Exchange collected data for patients 1-93 years of age. However, the reported proportion achieving target HbA1c refers specifically to those ≥18 years of age. iii. Australian site only included children <16 years of age. HbA1c = glycated haemoglobin, BP = blood pressure, LDL = low-density lipoprotein, TC = total cholesterol, T1DM/T1D = type 1 diabetes mellitus, ANDA = Australian National Diabetes Audit, T2DM = type 2 diabetes mellitus, UKNDA = United Kingdom National Diabetes Audit, US = United States
